# Treatment of ovarian cancer by targeting the tumor stem cell-associated carbohydrate antigen, Sialyl-Thomsen-nouveau

**DOI:** 10.18632/oncotarget.25289

**Published:** 2018-05-01

**Authors:** Kristen Starbuck, Linah Al-Alem, David A. Eavarone, Silvia Fatima Hernandez, Chiara Bellio, Jillian M. Prendergast, Jenna Stein, Daniel T. Dransfield, Bianca Zarrella, Whitfield B. Growdon, Jeff Behrens, Rosemary Foster, Bo R. Rueda

**Affiliations:** ^1^ Vincent Center for Reproductive Biology, Department of Obstetrics and Gynecology, Massachusetts General Hospital, Boston, MA, USA; ^2^ Siamab Therapeutics, Inc., Newton, MA, USA; ^3^ Division of Gynecologic Oncology, Vincent Department of Obstetrics and Gynecology, Massachusetts General Hospital, Boston, MA, USA; ^4^ Harvard Medical School, Boston, MA, USA

**Keywords:** ovarian cancer, sialyl-Tn, antibody-drug conjugate, cancer stem cell, tumor-associated carbohydrate antigen

## Abstract

Recurrent ovarian cancer (OvCa) is thought to result in part from the inability to eliminate rare quiescent cancer stem cells (CSCs) that survive cytotoxic chemotherapy and drive tumor resurgence. The Sialyl-Thomsen-nouveau antigen (STn) is a carbohydrate moiety present on protein markers of CSCs in pancreatic, colon, and gastric malignancies. We have demonstrated that human OvCa cell lines contain varying levels of cells that independently express either STn or the ovarian CSC marker CD133. Here we determine co-expression of STn and CD133 in a subset of human OvCa cell lines. Analyses of colony and sphere forming capacity and of response to standard-of-care cytotoxic therapy suggest a subset of OvCa STn^+^ cells display some CSC features. The effect of the anti-STn antibody-drug conjugates (ADCs) S3F-CL-MMAE and 2G12-2B2-CL-MMAE on OvCa cell viability *in vitro* and *in vivo* was also assessed. Treatment with S3F-CL-MMAE reduced the viability of two of three OvCa cell lines *in vitro* and exposure to either S3F-CL-MMAE or 2G12-2B2-CL-MMAE reduced OVCAR3-derived xenograft volume *in vivo*, depleting STn^+^ tumor cells. In summary, STn^+^ cells demonstrate some stem-like properties and specific therapeutic targeting of STn in ovarian tumors may be an effective clinical strategy to eliminate both STn^+^ CSC and STn^+^ non-CSC populations.

## INTRODUCTION

Ovarian cancer (OvCa) is the most lethal gynecologic cancer, with greater than 22,440 diagnoses and over 14,070 deaths projected in 2018 [1]. In large part, this is due to a lack of reliable early detection methods, resulting in greater than 75% of patients presenting with advanced stage disease. Optimal care of these patients begins with cytoreductive surgery, which alone is insufficient given the widespread nature of the disease and its propensity for extensive metastasis. Adjuvant taxane and platinum-based chemotherapy, therefore, is standard of care [2]. Unfortunately, despite aggressive surgery and adjuvant chemotherapy, most women with OvCa develop recurrent disease that is ineffectively treated with current therapies. Novel treatment strategies are urgently needed to target chemoresistant disease.

OvCa relapse can be explained in part by the persistence of a small subset of tumor cells often called cancer stem cells (CSCs) [3, 4] that are hypothesized to survive adjuvant chemotherapy despite a significant reduction in tumor burden. These CSCs are believed to possess the ability to self-renew as well as give rise to more differentiated tumor cells thereby recapitulating the heterogeneity of the original tumor and driving disease recurrence. CSCs are not unique to ovarian tumors and have been characterized in many solid tumors [5–8]. Accumulating research has revealed not only the importance of CSCs in tumor initiation, metastasis, recurrence, and chemoresistance, but also the potential of CSC-directed therapies to impact patient survival. Investigation of candidate targets has focused on cell membrane antigens such as MUC1, CD44v6, MUC4, MUC16 (CA-125), and other tumor-specific markers that are relatively specific for CSC populations [9–12]. Recently, glycan modifications of these proteins, among other carriers, have come into focus as a means to specifically target CSCs [13]. Lectin microarray analyses suggest that the sialylation and fucosylation state of CD133^+^ CSCs plays a role in their tumorigenicity [14, 15]. However, the branched, repeated structures and non-template driven synthesis of glycans, paired with the small population size of CSCs, makes it difficult to draw conclusions as to their full glycan profile given current glycomics technology [13, 16]. Understanding the specific carbohydrates altered on the surface of these CSCs would allow for more effective targeting of this population.

Aberrant glycosylation is a feature common to many cancers and leads to the formation of tumor-associated carbohydrate antigens (TACAs) [17, 18]. The TACA Sialyl-Thomsen-nouveau (also known as: STn, Sialyl-Tn or CD175s) is an O-glycan consisting of a sialic acid residue ɑ2,6-linked to GalNAcɑ-O-Ser/Thr. STn has been associated with cancer progression, lack of responsiveness to therapy, and poor prognosis [19–23]. Additionally, STn has been identified early in the transformation process and in pre-malignant disease in some cancers, suggesting that it may have a role in initiating tumorigenesis [21, 24]. Since STn expressing cancers are associated with increased tumor initiation, progression and chemoresistance, it is reasonable to hypothesize that STn is also present on CSC populations, especially since protein CSC markers such as MUC1 and CD44v6 are known carriers of STn [9–12]. Taken together, these findings suggest that the presence of STn on CSCs holds potential for the development of novel diagnostics as well as therapeutics.

We hypothesized that STn^+^ OvCa cells would demonstrate stem cell-like phenotypes compared to STn^-^ cells. Moreover, we postulated that utilizing a highly glycan-specific anti-STn ADC would reduce tumor burden in an *in vivo* model. Our results demonstrate that the STn antigen is expressed in OvCa cell lines and a subset of the STn^+^ cells co-express the OvCa CSC marker CD133. STn^+^ cells display a number of properties normally attributed to CSCs. More importantly, highly glycan-specific anti-STn antibodies conjugated to the cytotoxic drug monomethyl auristatin E (MMAE) as developed in Prendergast *et. al,* [25] decreased both OvCa cell viability *in vitro* and OvCa xenograft tumor volume *in vivo*, supporting our hypothesis that targeting of STn in ovarian tumors may be an effective clinical strategy.

## RESULTS

### STn and CD133 are co-expressed in ovarian cancer cell lines

**Figure 1:**
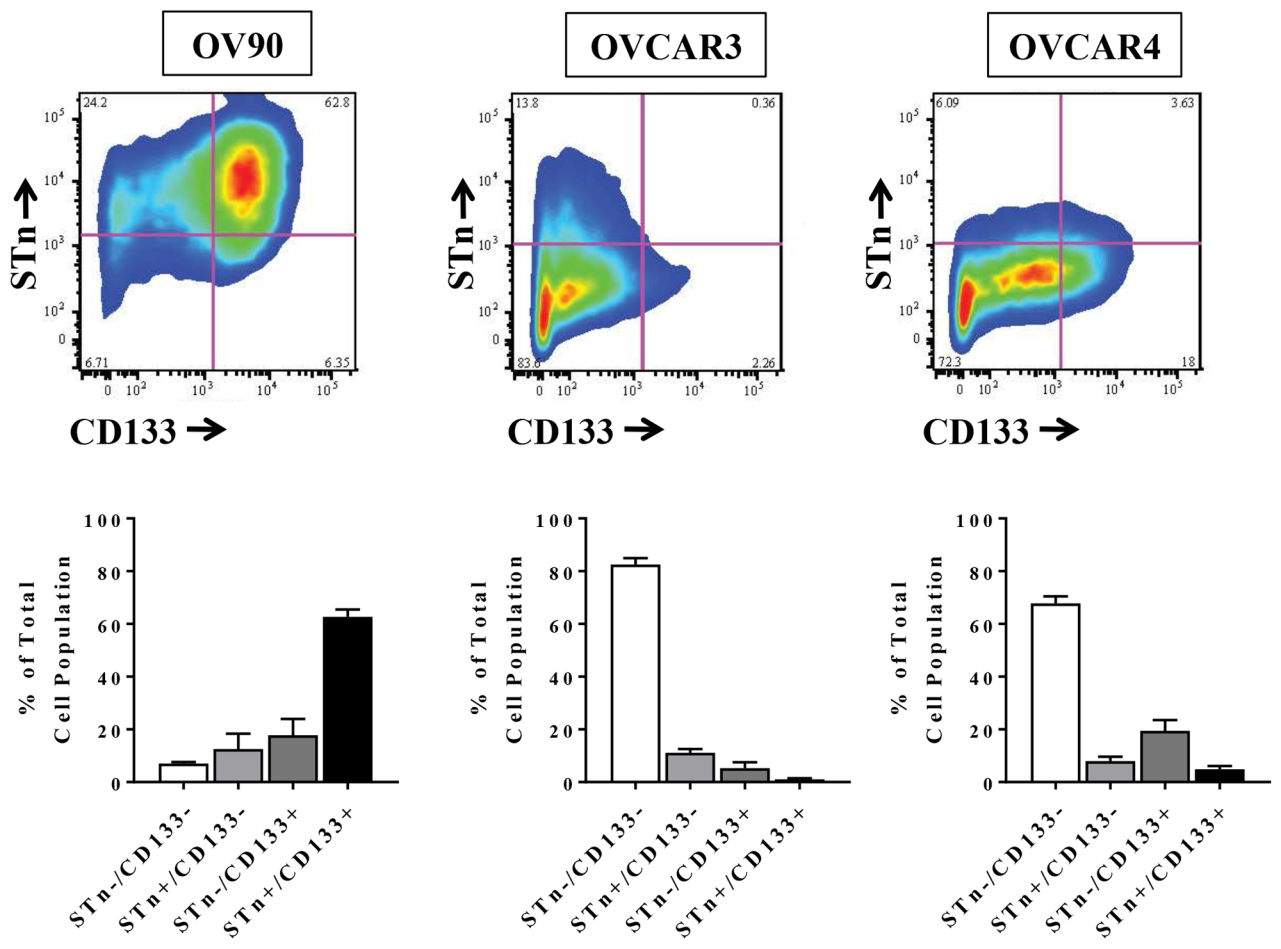
STn^+^ and CD133^+^ are co-expressed in human ovarian cancer cell lines A representative example of flow cytometric scatter plots for STn^+^ and CD133^+^ levels in OV90, OVCAR3 and OVCAR4 cells is shown along with quantification of the four populations analyzed (STn^-^/CD133^-^, STn^-^/CD133^+^, STn^+^/CD133^-^ and STn^+^/CD133^+^). Error bars represent the mean ± SEM of three independent experiments.

The ability to identify markers of CSCs is central to developing therapeutic strategies that target these cells and numerous studies have described different cell surface marker signatures that distinguish CSCs from the general population of tumor cells [3, 5–7]. To determine if STn is present on ovarian CSCs, we analyzed levels of STn and the known ovarian CSC marker CD133 in the established OvCa cell lines OV90, OVCAR3 and OVCAR4 (Figure 1). Flow cytometric analysis revealed that total CD133 levels were variable across all cell lines ranging from 6.29 ± 2.69 % to 80.45 ± 5.67%. Similarly, STn^+^ cells comprised 12.8 ± 2.99% to 75.14 ± 6.56% of the total cell population. We could readily detect cells that co-expressed STn and CD133 in each cell line at frequencies of 62.67 ± 2.77 % (OV90), 1.033 ± 0.41% (OVCAR3) and 4.88 ± 1.24% (OVCAR4). These observed ranges of CD133 and STn levels are consistent with previously reported data [26–31].

### STn^+^ and CD133^+^ cells have increased colony formation capacity

**Figure 2:**
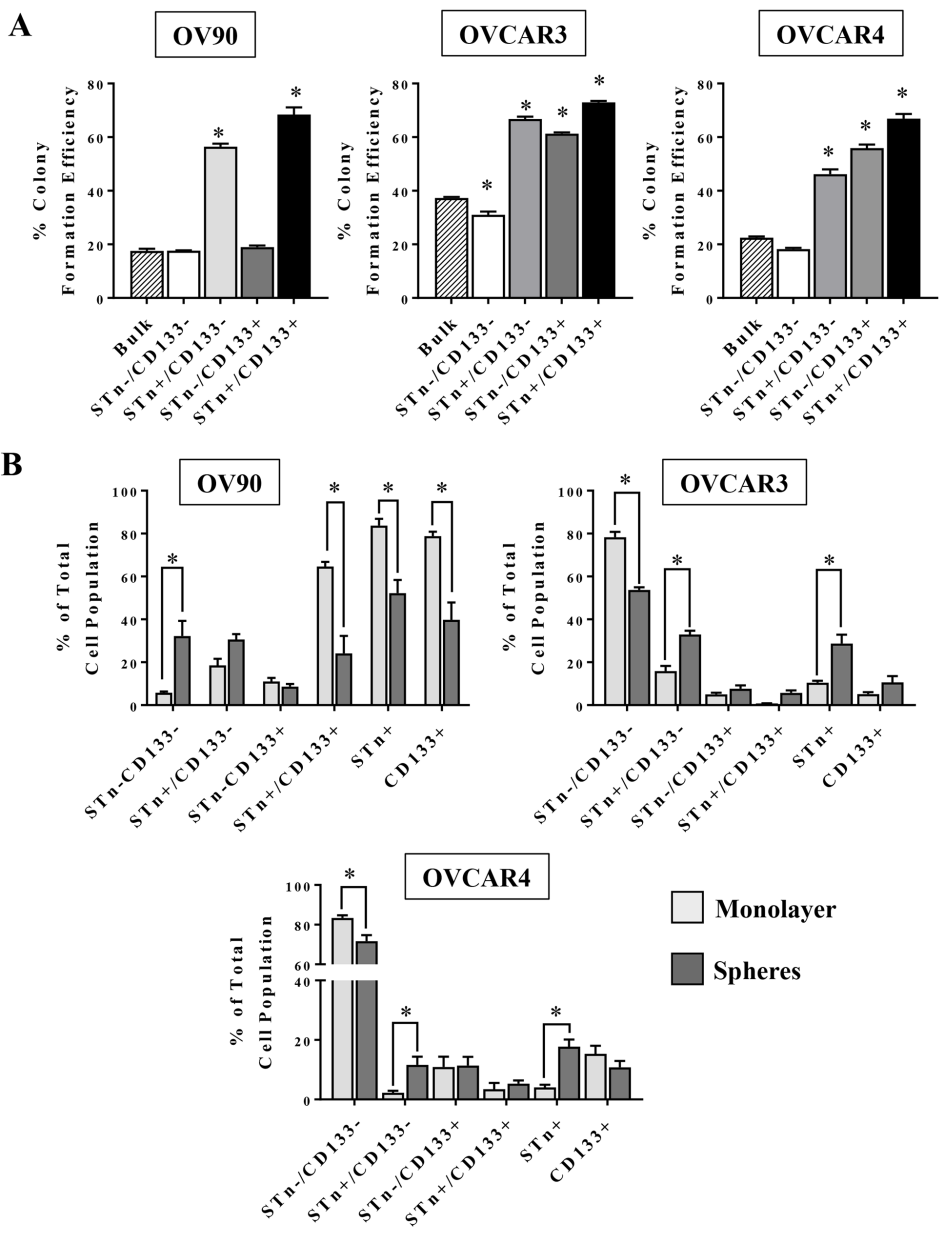
STn^+^ and CD133^+^ cells have enhanced colony formation capacity and are enriched in spheres **(A)** The indicated sub-populations of OV90, OVCAR3 or OVCAR4 cells were isolated by FACS and seeded in soft agar. Unsorted OV90, OVCAR3 or OVCAR4 cells were seeded in parallel as a control. After 21 days, colonies ≥20 cells were counted and the percent colony formation efficiency of each population was calculated as described in Material and Methods and compared to that of unsorted cells. **(B)** OV90, OVCAR3 and OVCAR4 cells were cultured under either monolayer or sphere conditions for 10-12 days. At the end of this incubation period, the relative frequency of each sub-population was determined by flow cytometry. Error bars represent the mean ± SEM of a minimum of three independent experiments ^*^p < 0.05.

Given the variable presence of STn in OvCa cells and its propensity to be co-expressed on some CD133^+^ cells, a series of analyses were initiated to investigate whether STn^+^ cells have CSC-like properties. The capacity for anchorage-independent growth is a hallmark of cell transformation and correlates with tumorigenicity *in vivo* [32]. OVCAR3, OVCAR4 and OV90 cells were sorted into STn^-^/CD133^-^, STn^+^/CD133^-^, STn^-^/CD133^+^ and STn^+^/CD133^+^ fractions and were plated in soft agar to determine the capacity of each population to form colonies relative to that of unsorted cells (Figure 2A). In all cell lines, STn^+^/CD133^-^ and STn^+^/CD133^+^ cells had increased (p < 0.05) colony formation capacity compared to the bulk population. In contrast, despite the OVCAR3 and OVCAR4 STn^-^/CD133^+^ cells displaying an increased colony forming capacity the OV90 STn^-^/CD133^+^ cells remained no different at forming colonies when compared to the bulk population. Interestingly, the unsorted bulk population had limited colony forming capacity, suggesting that the STn enriched populations have enhanced colony forming capacity.

### STn^+^ cell frequency is increased in spheres

Tumorsphere culture is an *in vitro* model that has been shown to enrich for cells with stem-like phenotypes [33, 34]. We analyzed the relative frequency of STn^+^ and CD133^+^ cells in OV90, OVCAR3 and OVCAR4 cells cultured in either standard monolayer two dimensional conditions or three-dimensional tumorsphere conditions. In the OVCAR3 and OVCAR4 cell lines, STn^+^/CD133- and STn^+^ cell frequency was increased in tumorspheres compared to cells cultured as a monolayer (Figure 2B). Concurrently, there was a decrease in viable STn^-^/CD133^-^ cells in the spheres implying that in these two lines STn^+^ cells were better able to withstand the serum free culture conditions necessary for sphere formation. More dramatic, however, was the contrasting changes observed in the OV90 cell line. Culturing OV90 in the monolayer and sphere conditions optimized for OVCAR3 and OVCAR4 experiments resulted in reductions (p<0.05) in the total STn^+^, total CD133^+^ cells and the STn^+^/CD133^+^ cells under the sphere conditions. In addition, there was an increase in the STn^-^/CD133^-^ cell fraction. It is not clear why the OV90 cell line, which is inherently high in CD133^+^ and STn^+^ cells, did so poorly in the sphere conditions optimized for OVCAR3 and OVCAR4 unless OV90 cells required more growth factors than supplied in order to maintain their survival properties.

### STn^+^ and CD133^+^ cells persist following cytotoxic chemotherapy

**Figure 3:**
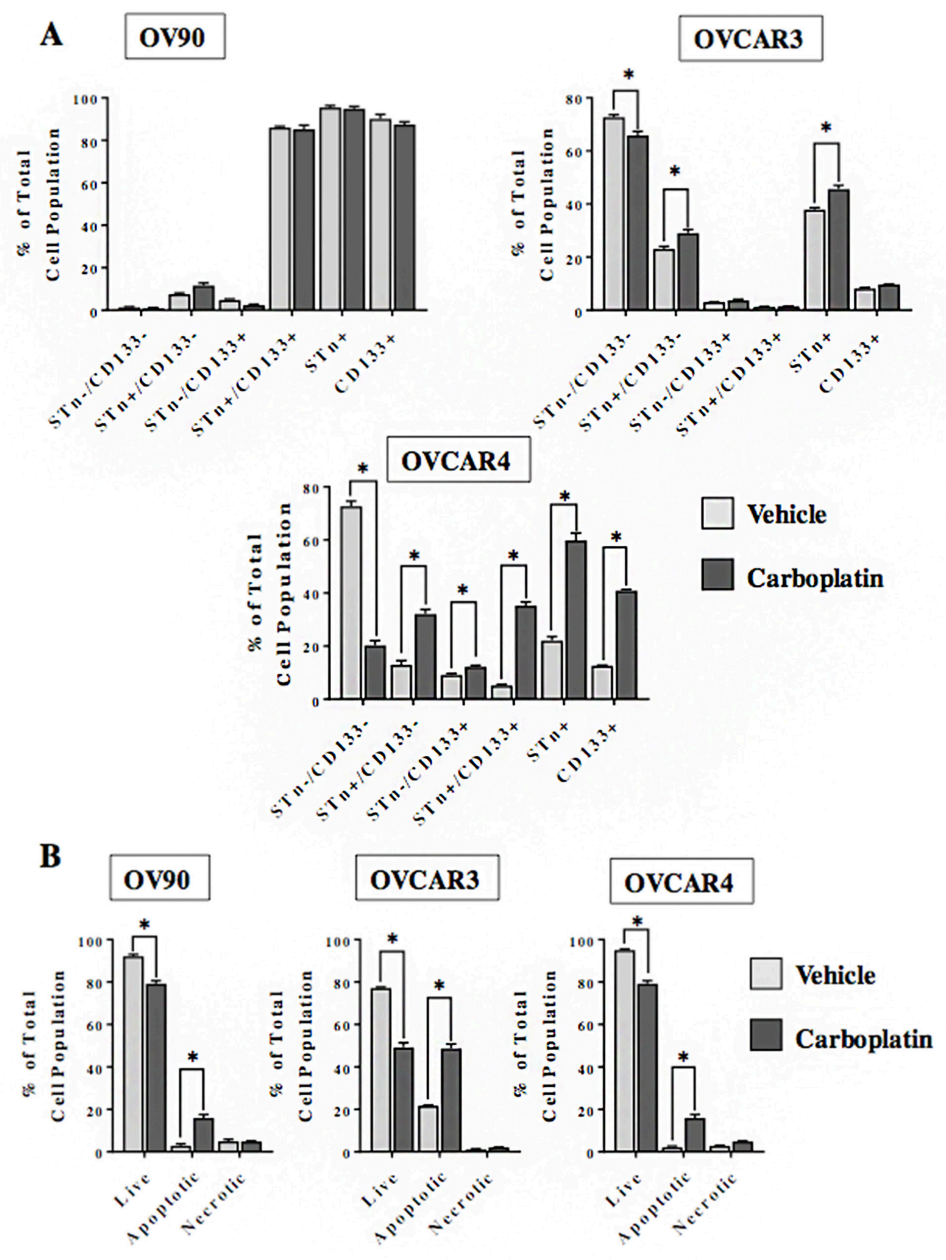
STn^+^ and CD133^+^ cells display chemoresistance **(A)** OV90, OVCAR3 and OVCAR4 cells were cultured for 72 hours in the presence of either vehicle control or carboplatin (10 μM). Following treatment, adherent cells were trypsinized and analyzed by flow cytometry to determine the relative frequency of STn^-^/CD133^-^, STn^+^/CD133^-^, STn^-^/CD133^+^ and STn^+^/CD133^+^ sub-populations. **(B)** In a parallel analysis, the total number of live, apoptotic and necrotic cells remaining after 72 hours of exposure to vehicle control or carboplatin (10 μM) was determined by annexin assay. Error bars represent the mean ± SEM. ^*^p < 0.05. All experiments were repeated a minimum of three times.

One notable feature of CSCs is their relative resistance to chemotherapy. We investigated the effect of *in vitro* treatment with carboplatin on the frequency of STn^+^ and CD133^+^ sub-fractions in OVCAR3, OVCAR4 and OV90 cells (Figure 3). With the exception of OV90, carboplatin treatment led to a higher (p<0.05) frequency of STn^+^ and STn^+^/CD133^-^ cells as compared to the vehicle-treated control. Interestingly, the STn^+^/CD133^+^ and CD133^+^ cell fractions derived from the OVCAR4 cells were higher (p<0.05) relative to the vehicle treated controls. In contrast, the CD133+ enrichment was not observed in the OVCAR3 cells post 72 hours of treatment. Analysis of cell viability determined that the number of live cells is reduced (p<0.05) in OV90, OVCAR3 and OVCAR4 following exposure to carboplatin. The proportion of STn^-^/CD133^-^ cells is similarly decreased in OVCAR3 and OVCAR4 lines. While there was a modest decrease (p<0.05) in OV90 cell viability there was no difference in the STn^-^/CD133^-^ cells between the carboplatin and vehicle-treated cells. This is likely due in part to the small number of double negative cells in the OV90 line. Taken together, these data suggest that in OVCAR3 and OVCAR4 cell lines the STn^-^/CD133^-^ cell fractions are particularly sensitive to the cytotoxic effects of carboplatin. The observed enrichment in STn^+^ and CD133^+^ sub-fractions following exposure to carboplatin indicate that those cells are relatively chemoresistant. The minimal impact of carboplatin on the OV90 cell line in which the frequencies of STn^+^ and CD133^+^ are inherently high indirectly supports this concept.

### An antibody drug conjugate (ADC) targeting STn^+^ cells effectively decreases ovarian cancer cell viability *in vitro*

**Figure 4:**
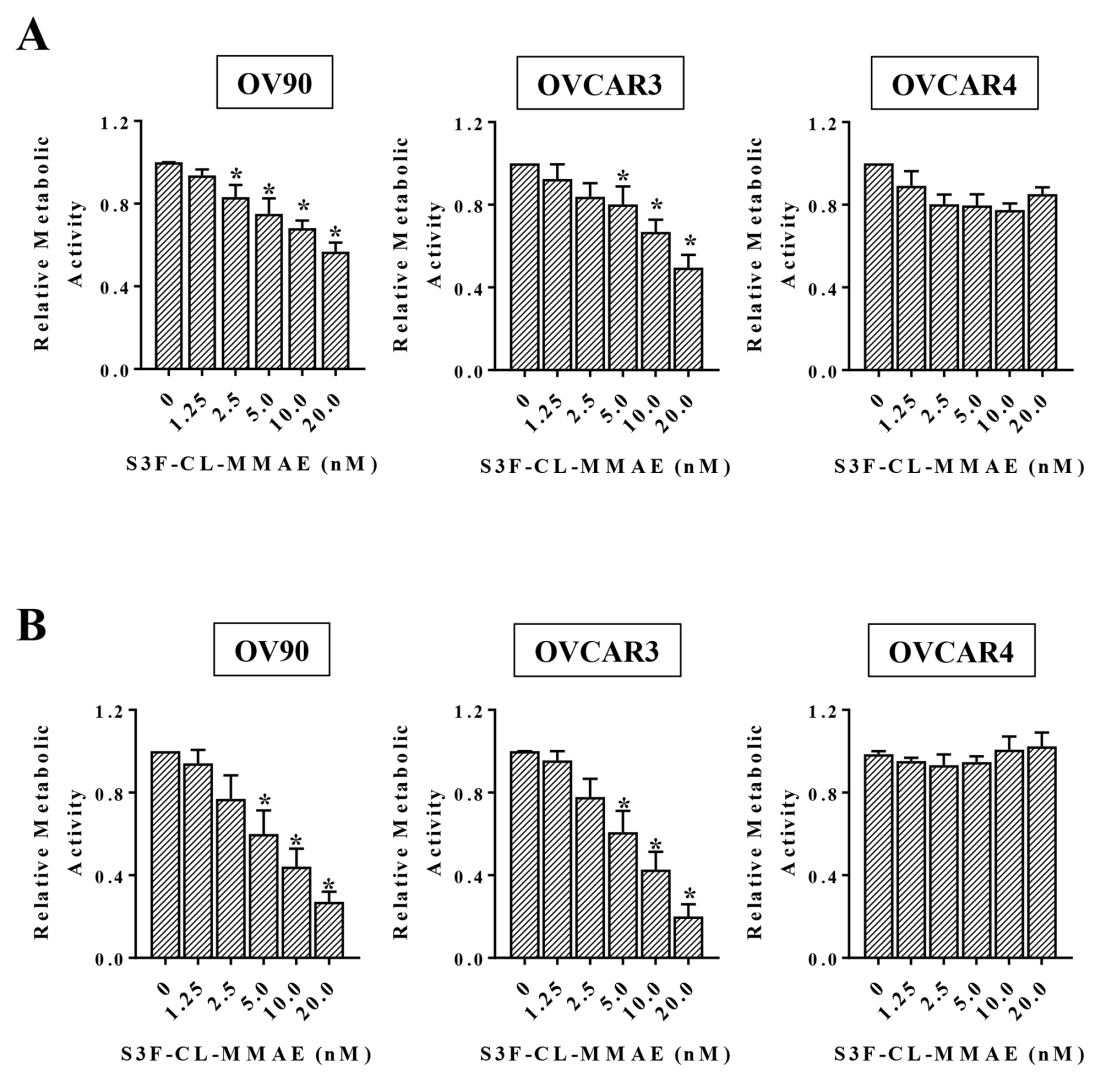
Anti-STn ADC decreases cell viability *in vitro* The indicated cell lines were treated in quadruplicate with increasing concentrations of S3F-CL-MMAE for either 72 **(A)** or 144 hours **(B)**. The effect of S3F-CL-MMAE on metabolic activity was determined by MTT assay and assessed relative to the untreated control. Error bars represent the SEM. ^*^p <0.05. All experiments were repeated four times.

Since our analyses suggest that both STn^+^/CD133^-^ and STn^+^/CD133^+^ OvCa cells exhibit some stem cell like characteristics, we sought to target STn-positive cells by exploiting our novel specific anti-STn antibody [25]. We developed a cathepsin B-labile maleimidocaproyl-valine-citruline-p-aminobenzyloxycarbonyl-monomethyl auristatin E ADC (MC-vc-PAB-MMAE, referred to as CL-MMAE in the text). This is the same payload system incorporated in Adcetris^®^, an FDA-approved drug for the treatment of Hodgkin lymphoma and systemic anaplastic large cell lymphoma [35, 36]. We assessed the effect of the anti-STn ADC S3F-CL-MMAE on the metabolic activity of OvCa cell lines with varying levels of STn following 3 days (Figure 4A) or 6 days (Figure 4B) of treatment. In OV90 and OVCAR3 cells, exposure to 20 nM S3F-CL-MMAE for 3 days decreased metabolic activity by approximately 40 % (Figure 4A). A longer 6 day treatment reduced the IC_50_ for S3F-CL-MMAE to 5-10 nM in both OV90 and OVCAR3 (Figure 4B), demonstrating a positive correlation between cytotoxicity and duration of treatment in these cell lines. OVCAR4 cells were largely resistant to both the 3 day and 6 day S3F-CL-MMAE treatment over the tested 0 nM to 20 nM concentration range. We have determined that OVCAR4 cells are sensitive to 3 day treatment with higher concentrations (50-100 nM) of S3F-CL-MMAE (data not shown).

### Anti-STn ADC treatment *in vitro* decreases CSC frequency

**Figure 5:**
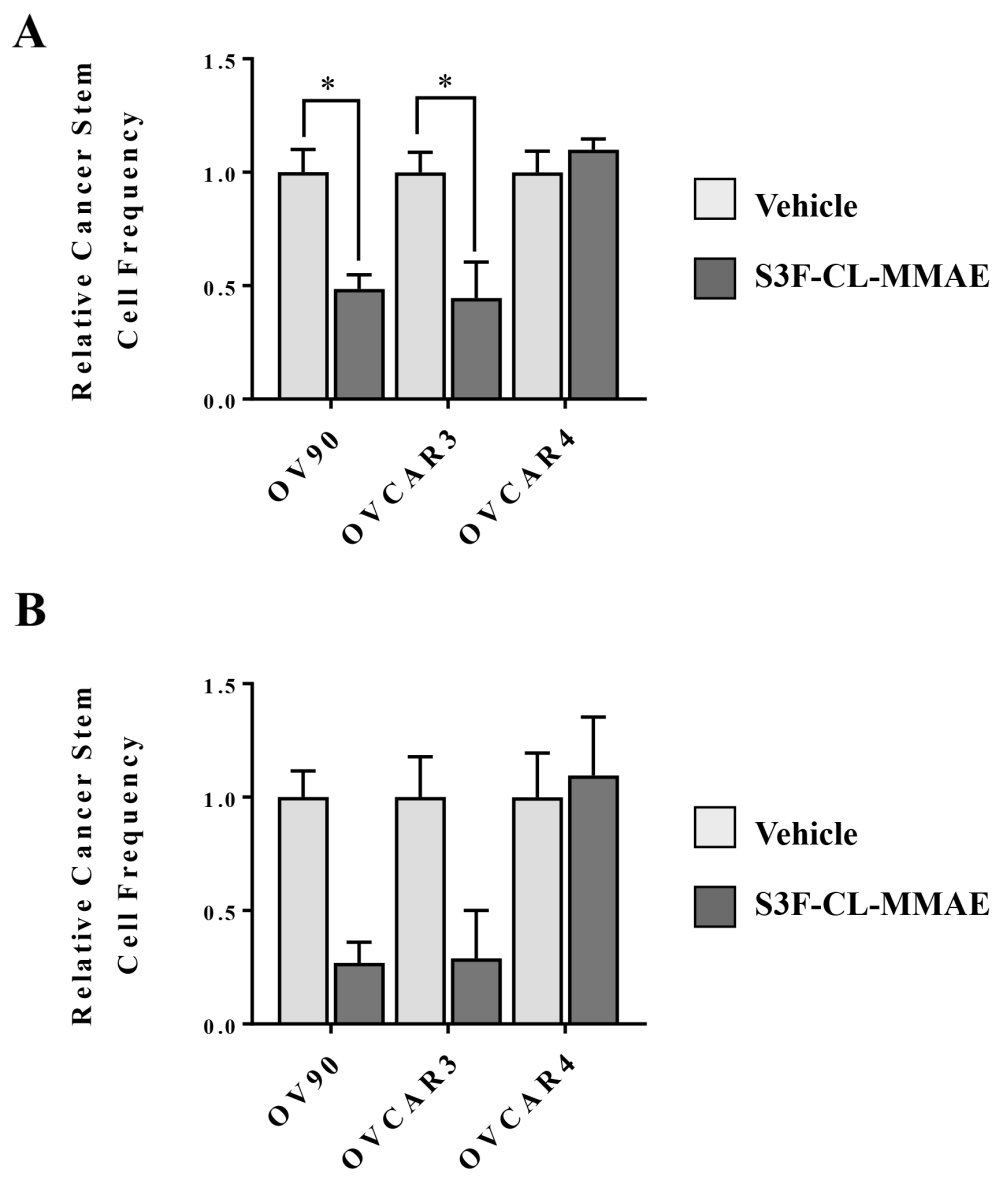
S3F-CL-MMAE decreases CSC frequency *in vitro* The extreme limiting dilution assay was used as surrogate to determine the effect of S3F-CL-MMAE on CSC frequency. Seventy two **(A)** and 144 hours **(B)** following treatment with vehicle or 10 nM S3F-CL-MMAE, OV90, OVCAR3 and OVCAR4 cells (1-10-20 or 40/well) were plated under tumorsphere culture conditions and the number of spheres that formed after 7-8 days in culture was determined. Stem cell frequencies were calculated as described in Materials and Methods. Error bars represent the SEM. ^*^p < 0.05. All experiments were repeated four times.

Targeting CSCs is predicted to eliminate chemoresistant tumor cell populations and block the development of recurrent disease driven by the tumor initiating capacity of CSCs. Since definitive identification of CSCs is challenging, the extreme limiting dilution assay (ELDA) is often utilized as a surrogate method for determining the approximate CSC frequency in a population of cancer cells [37]. The ELDA assesses the functional capacity of cells to form tumorspheres in serum-free non-adherent conditions at extremely low cell numbers. In our analysis, OV90, OVCAR3, OVCAR4 cells treated were treated with either vehicle control or 10 nM S3F-CL-MMAE or vehicle control. At the end of the 3 or 6 day incubation period, any remaining cells were cultured under the appropriate conditions and tumorsphere formation was monitored (Figure 5). In OV90 and OVCAR3 cells, exposure to S3F-CL-MMAE decreased the relative CSC frequency by approximately 50% in S3F-CL-MMAE treated cells after 3 days relative to their vehicle treated counterparts. While the trend was similar in the cells assessed 6 post treatment it did not reach significance. Parallel analyses in OVCAR4 showed no difference in the CSC frequency between the vehicle control and S3F-CL-MMAE treated cells (1.0 and 1.13, respectively). This observation is consistent with our finding that OVCAR4 cells are largely resistant to 10 nM S3F-CL-MMAE.

### Anti-STn-ADC treatment impact on CD133 and STn populations *in vitro* varies with time and cell type

**Figure 6:**
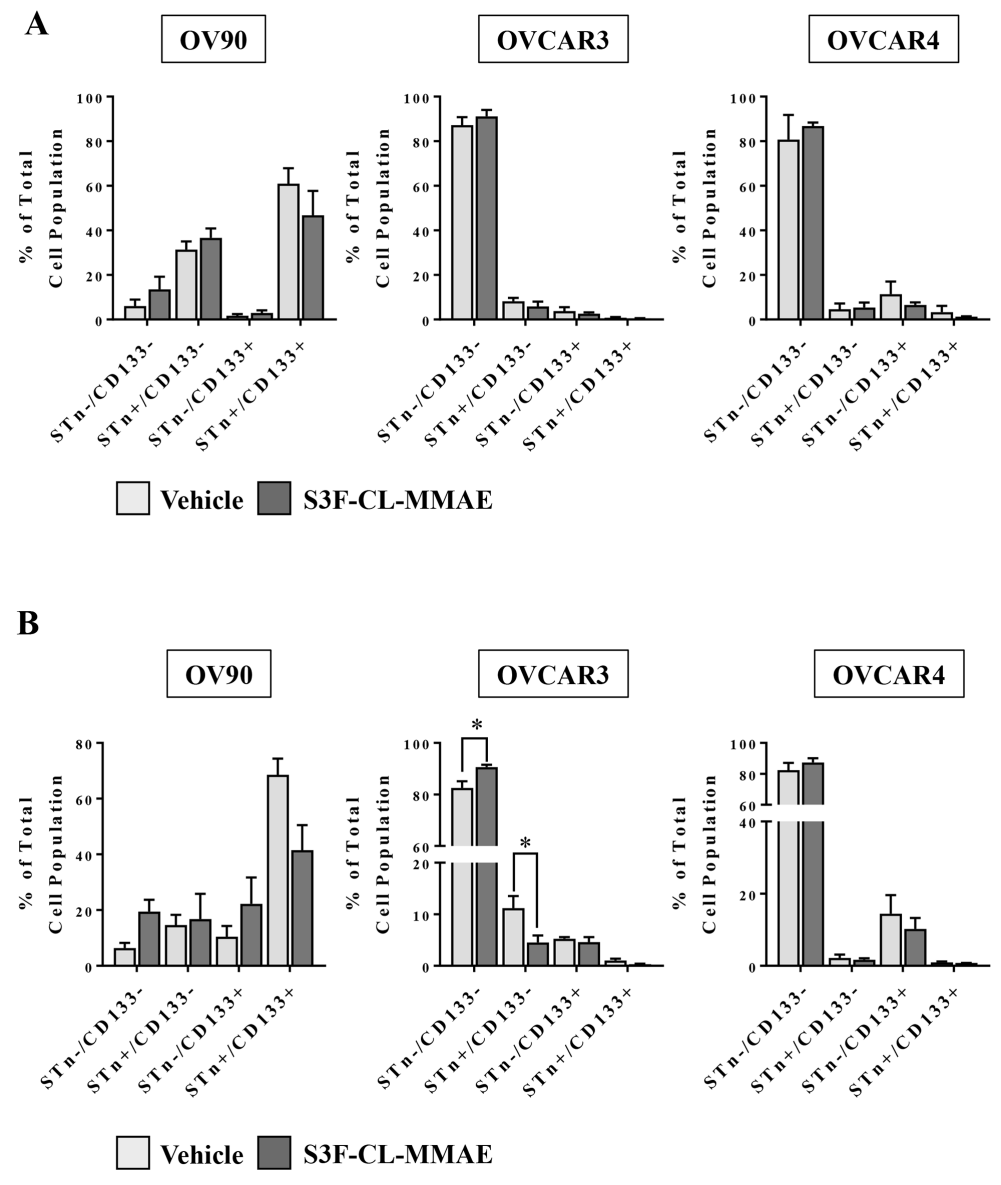
Treatment with the anti-STn, S3F-CL-MMAE, on CD133 and STn populations *in vitro* OV90, OVCAR3 and OVCAR4 cell lines were harvested 72 **(A)** or 144 **(B)** hours post treatment with vehicle or S3F-CL-MMAE (10 nM), and the relative frequency of STn^-^/CD133^-^, STn^+^/CD133^-^, STn^-^/CD133^+^ and STn^+^/CD133^+^ fractions was determined by flow cytometry. Error bars represent the mean and SEM. ^*^p < 0.05. All experiments were repeated four times.

After observing a S3F-CL-MMAE -induced decrease in tumorsphere formation in OV90 and OVCAR3 cells, a secondary experiment was performed to determine if the drug preferentially impacted cell fractions. OV90 and OVCAR3 cells were collected 3 or 6 days post treatment with vehicle or S3F-CL-MMAE (10nM), STn^-^/CD133^-^, STn^+^/CD133^-^, STn^-^/CD133^+^ and STn^+^/CD133^+^ fractions flow cytometry and analyzed (Figure 6A, 6B). Statistical difference was only evident in the OVCAR3 cells at the 6 day time point whereby the STn^-^/CD133^-^ cell fraction increased in response to treatment with S3F-CL-MMAE when compared to vehicle (Figure 6B). In contrast, the STn^+^/CD133^-^ decreased following treatment with S3F-CL-MMAE when compared to vehicle (Figure 6B). A similar trend was observed in the OV90 cells at the same time point but the shift did not reach significance (Figure 6B). As anticipated there was no significant change in the cell fractions in the OVCAR4 cells at either time point given the known lack of impact at this concentration of S3F-CL-MMAE (Figure 6A, 6B). These data support the concept that S3F-CL-MMAE is preferentially impacting STn populations, albeit likely in a dose and time point dependent manner.

### Anti-STn ADC treatment impedes tumor growth *in vivo*

**Figure 7:**
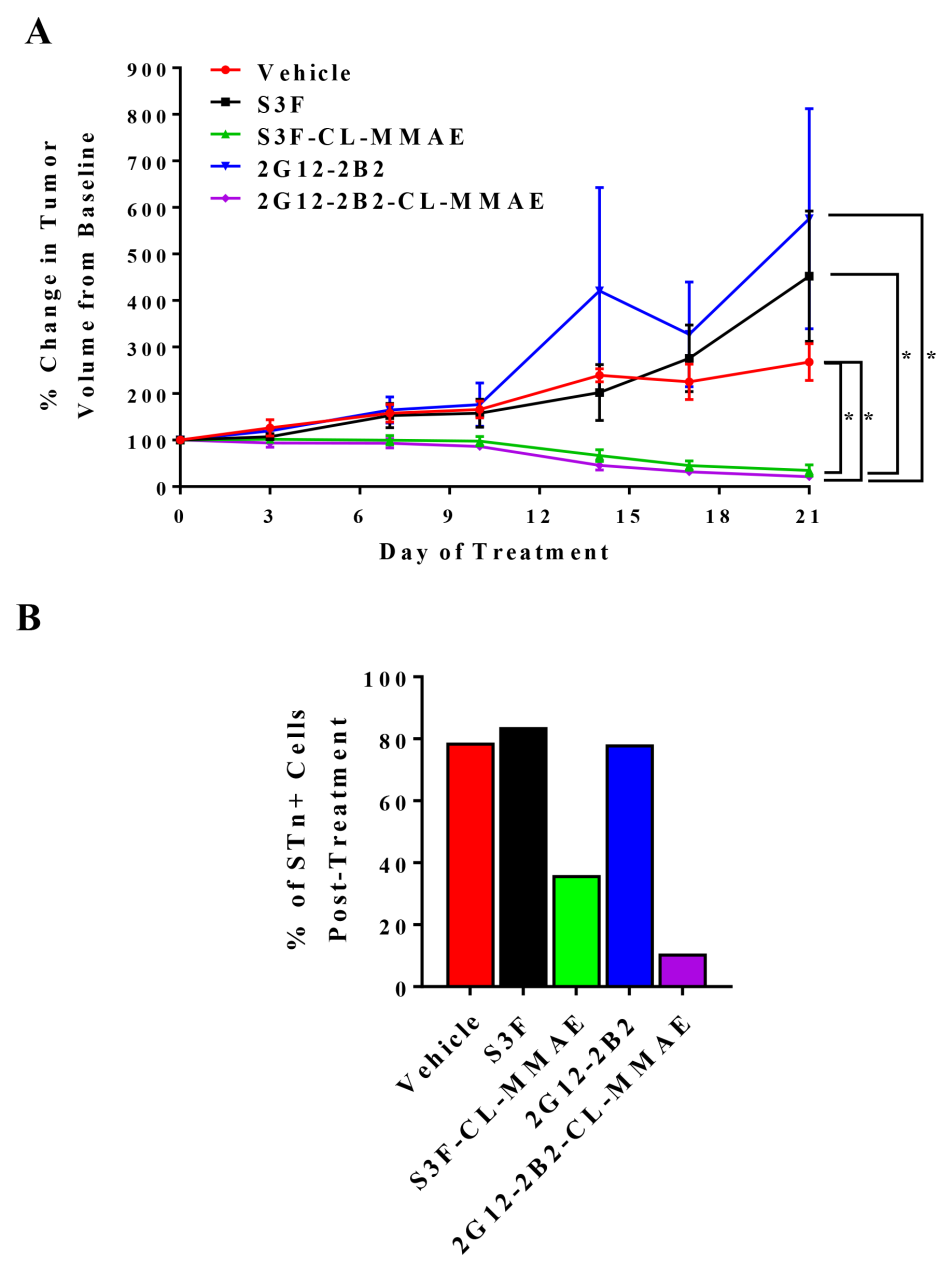
Anti-STn ADCs impede OvCa xenograft growth *in vivo* and target STn^+^ cells **(A)** Mice bearing tumors derived from OVCAR3 were treated with vehicle, unconjugated S3F, S3F-CL-MMAE, unconjugated 2G12-2B2, or 2G12-2B2-CL-MMAE. Tumors were measured twice weekly, and the percentage change in tumor volume compared to the baseline volume (100%) is shown. Error bars represent the mean ± SEM ^*^p < 0.05. **(B)** Xenografts collected at the end of the *in vivo* treatment were examined to assess the relative frequency of STn^-^/CD133^-^, STn^+^/CD133^-^, STn^-^/CD133^+^ and STn^+^/CD133^+^ sub-populations.

To evaluate potential anti-tumor efficacy of our anti-STn ADCs *in vivo*, we established OVCAR3 xenografts in NOD/SCID mice and analyzed the ability of two different anti-STn ADCs (S3F-CL-MMAE and 2G12-2B2-CL-MMAE) to impact tumor growth. We also evaluated the efficacy of the corresponding non-conjugated anti-STn antibodies as a control. At the completion of treatment, both S3F-CL-MMAE and 2G12-2B2-CL-MMAE induced significant tumor regression as compared to the vehicle control (p<0.05) confirming successful single-agent targeting of OvCa xenografts (Figure 7A). Each anti-STn ADC was significantly more effective at impeding tumor growth than its non-conjugated counterpart (p<0.05). Importantly, no significant changes in mouse weight were observed during treatment (Supplementary Figure 1) and no other toxicities were noted.

To assess whether the anti-tumor effects were specific to STn^+^ cell targeting by the anti-STn ADCs and not due to generalized distribution of MMAE, xenografts were harvested 6 hours following administration of the last dose in each treatment group and the level of STn was assessed by flow cytometry (Figure 7B). It should be noted that the tumors exposed to S3F-CL-MMAE or 2G12-2B2-CL-MMAE were so small the remaining tumor tissue had to be pooled to have adequate events to measure. In tumors treated with vehicle or either unconjugated anti-STn antibody, STn levels remained high (78.2-83.8% of the total cell population) after the 28 day treatment period. In contrast, chronic treatment with S3F-CL-MMAE or 2G12-2B2-CL-MMAE reduced the proportion of STn+ cells to 36.0% and 10.7 % respectively suggesting an on-target treatment effect. Finally, Ki67 immmunohistochemical analysis of the harvested tumors demonstrated that both anti-STn ADCs reduced the proliferative index in the chronically-treated xenografts (Supplementary Figure 2). This effect was not observed in xenografts treated with the vehicle or either unconjugated anti-STn antibody.

## DISCUSSION

The clinical challenge for women diagnosed with OvCa continues to be the resurgence of chemoresistant tumor cells that are relatively impervious to most current available treatment options. Research efforts are often focused on the development of single agent or combination therapies that can impact platinum resistant disease. Preferably, this would comprise treatment strategies that induce a durable remission through total eradication of all tumor cell populations or maintenance therapies that can hold tumor resurgence at bay for a longer period without excessive toxicity. Here we have confirmed that STn is present in OvCa and holds promise for the development of therapeutic treatment modalities. Collectively, our data provides evidence to suggest that targeting the STn antigen on OvCa cells with an anti-STn ADC can selectively inhibit tumor cell proliferation and induce tumor cell death in both *in vitro* and *in vivo* models.

The surface of vertebrate cells are modified with a wide variety of glycans with considerable structural diversity [39], forming a dense glycocalyx at the cellular surface. Altered glycosylation can be indicative of malignancy, and STn is known to be present in more than 80% of human carcinomas [21]. Others have generated anti-STn antibodies and demonstrated variable percentages of STn positive cells in primary tumors by IHC (for a comprehensive review see Julien et al. [21]). Of note, the historical anti-STn antibody B72.3 conjugated to Indium-111 was the first to be approved for imaging studies by the FDA in 1992 and was reportedly used to detect OvCa with a sensitivity of 85% in primary OvCa and 84% in abdominal metastasis with no adverse effects/false localizations reported [38].

Glycans are not directly encoded in the genome like RNA or protein, but are rather generated by multiple glucosidases and glycosyltransferases whose expression and activity can be altered by both the intra- and extracellular environment [40]. The interplay of these enzymes generates the cellular surface glycans which can act as a unique identifier, distinguishing cell populations within an organism. Global alterations of the surface glycome have been demonstrated to occur during development, differentiation [41] and malignant transformation [21]. Surface glycosylation of induced pluripotent stem cells, embryonic stem cells and CSCs has also been reported to be unique from surrounding differentiated somatic cells [40]. Some well-known pluripotency markers such as stage-specific embryonic antigens (Lewis X/SSE-1 and SSEA3-4) along with tumor rejection antigens (TRA-1-60 and TRA-1-81) are glycan epitopes [42].

Understanding the interface of aberrant STn surface glycosylation and CSCs could prove to be advantageous in treating primary disease and possibly preventing disease recurrence. In some human CSCs, CD44 and MUC1 are major carriers of the STn antigen [17, 43]. Tumor-associated MUC1 is characterized by altered glycosylation such as hypoglycosylation and increased sialylation relative to that of MUC1 on some normal epithelial cells where it serves a barrier function. Our data suggest that the STn detected on some CSCs is the same as that observed on non stem cancer cells and could therefore serve as a target for therapeutics and/or diagnostics [44]. Other investigators have similarly determined that altered N-linked glycans can influence stem properties in ovarian and pancreatic cells [45].

In earlier studies, we demonstrated that STn levels vary widely across established OvCa cell lines [25] likely due to heterogeneity among the evaluated lines. In analogous analyses, we and others have reported similar variability in expression of the known CSC marker CD133 [28, 46]. Given the possible role of altered glycosylation in CSC function, we initially assessed potential co-expression of CD133 and STn and determined that all examined cell lines had varying levels of STn^+^/CD133^+^ cells. This range is not entirely unexpected since CD133 is not the only marker for CSCs in OvCa [3, 4]. OvCa STn^+^ cells display many properties shared by CD133^+^ cells, which are recognized as CSCs in other tumor types [4, 28, 47]. STn^+^/CD133^-^ cells, like STn^-^/CD133^+^ cells, demonstrated increased colony formation capacity, were enriched under culture conditions that promoted tumorsphere formation and persisted after exposure to cytotoxic chemotherapeutics. Significantly, pre-treatment with the anti-STn ADC S3F-CL-MMAE in an extreme limiting dilution assay dramatically reduced the sphere formation capacity of OVCAR3 and OV90 cells suggesting direct targeting of STn^+^ cells impacts cancer stem cell frequency. Currently, it is not clear if this decrease in CSC number can be enhanced by longer exposure to the anti-STn ADC. Alternatively, it is possible that those cells successfully evading the killing effects of S3F-CL-MMAE do not express STn but are ovarian CSCs marked by CD133, CD117 and/or ALDH expression and/or activity.

Despite the overwhelming evidence that TACAs may serve as viable targets and/or biomarkers of malignancy, there has been limited progress in developing an effective clinical application. Limited clinical trials using the second generation B72.3 antibody CC49 [48] demonstrated patient efficacy. Twenty-seven OvCa patients who failed first line chemotherapy were administered ^177^Lu-CC49. Patients in this Phase I/II study experienced a >50% tumor reduction and two of nine patients remained recurrence free at >6 to 35 months with marrow suppression as the dose-limiting toxic effect of this IP therapy [49, 50]. The most well documented STn vaccine was Theratope (BioMira). In mouse studies, Theratope immunization induced a potent antibody response that was shown to mediate a delay in the growth of injected STn-expressing mammary carcinoma cells [51]. Unfortunately, Theratope failed to meet its primary endpoint in a Phase III clinical trial in metastatic breast cancer [52]. STn expression in breast cancer is highly heterogeneous between patients, ranging from 25%-80% depending on the study and detection method but no such stratification as performed on Theratope patients [21]. A *post hoc* analysis stratifying patients found that a subset of patients receiving hormonal therapy showed a statistically significant increase in median overall survival of 7.5 months when treated with Theratope compared to hormone therapy alone. Those women on endocrine therapy who had a median or greater antibody response to the STn vaccine had significantly longer median overall survival than those who had a below-median antibody response, supporting the therapeutic potential of targeting STn in particular patient populations [53]. This *post hoc* analysis suggests the Theratope trial could have been more successful if they incorporated the use of a companion diagnostic to pre-select patients based on STn expression prior to enrollment. Importantly, however, Theratope was well tolerated with minimal toxicity, demonstrating the safety of targeting STn for cancer therapy.

Our finding that targeting STn^+^ cells *in vivo* with a highly specific antibody conjugated to auristatin resulted in marked decreases in tumor burden without any obvious toxicity suggests that an anti-STn ADC approach may serve as a viable option in eliminating non-CSC as well as some CSC populations. On-target effects were confirmed by flow cytometric analysis of the residual tumor which revealed marked reduction in STn^+^ cells in the anti-STn ADC treated xenografts. This study was not designed to assess durability and the impact of long-term anti-STn ADC treatment remains to be determined. Regardless, our results provide evidence the STn^+^ fraction likely contains some cells with CSC properties. Our studies suggest that either combination or sequential coupling of anti-STn therapy with conventional cytotoxics would target both bulk tumor cells and CSCs that carry the STn antigen to induce a more durable remission without excessive toxicity in OvCa patients.

## MATERIALS AND METHODS

### Cell culture

Ovarian cancer cell lines OVCAR3 and OV90 were obtained from ATCC and OVCAR4 was generously provided by Dr. Panos Konstantinopoulos, MD, PhD (Dana Farber Cancer Institute, Harvard Medical School, Boston MA). These cell lines have been characterized previously [54–56]. Established cell lines were subjected to human cell identity verification (STR profiling) at Dana Farber Cancer Institute (http://moleculardiagnosticscore.dana-farber.org). All cell lines were regularly tested for mycoplasma contamination.

OV90 cells were cultured in a 1:1 mixture of MCDB 105 medium (Cell Applications, San Diego, CA) containing a final concentration of 1.5 g/L sodium bicarbonate and Medium 199 (Gibco, Gaithersburg, MD) containing a final concentration of 2.2 g/L sodium bicarbonate and 20% FBS [54–56]. OVCAR3 cells were cultured in RPMI 1640 medium supplemented with 10% fetal bovine serum (FBS), 1% penicillin- streptomycin (Life Technologies, Grand Island, NY), and 0.01 mg/ml bovine insulin (Sigma-Aldrich, Natick, MA). OVCAR4 cells were cultured in RPMI 1640 supplemented with 10% fetal bovine serum (FBS) and 1% penicillin- streptomycin (Life Technologies). All cell lines were maintained at 37°C in 5% CO_2_.

### *In vitro* treatment of OvCa cell lines with carboplatin

Carboplatin was purchased from Sigma Aldrich (St. Louis, MO, USA) and a stock of 10 mg/mL was prepared in dH_2_O and stored at room temperature. OVCAR3, OVCAR4 and OV90 cell lines were plated in 6-well plates (1 × 10^5^ cells/well) in complete media. After overnight incubation, cells were treated with carboplatin (10 μM) for 72 hours and cell viability and CD133 and STn levels were evaluated by flow cytometry.

### Flow cytometry

Flow cytometry was used to assess subpopulations of OvCa cells. Following trypsinization and incubation with FcR blocking reagent (Miltenyi Biotec), cells were stained with anti-CD133/2 (phycoerythrin (PE) conjugated; Miltenyi Biotec) and anti-STn antibody S3F (Siamab Therapeutics, Inc., Newton, MA) [25] directly conjugated to Alexa Flour 647 using the Zenon antibody labeling kit (Thermo Fisher Scientific, Waltham, MA). Live-Dead Fixable Dead Cell Stain kit (Invitrogen) was added to exclude non-viable cells. Cell viability was assessed using Annexin/PI staining kit (Roche, Basel, Switzerland): a volume of 100 μL buffer was added to resuspend cells followed by adding 1.5 μL of annexin V-FITC and 1.5 μL of PI staining reagents. After an incubation at 25°C for 15 minutes in the dark, the live, apoptotic and necrotic cells were analyzed by flow cytometry. After washing, cells were fixed in 4% paraformaldehyde for 20 minutes and analyzed using LSRII (BD Biosciences, San Jose, CA) within 12 hours. Data were analyzed using FlowJo software (version 10.0.8). For analysis of cells derived from xenograft tumors, cells were dissociated to a single-cell suspension and stained with a FITC-conjugated H^2^K^d+^ (BD Biosciences) antibody to allow exclusion of murine cells.

### Soft agar assay

Six well plates were coated with 1.2% agar and stored at 4°C. OVCAR3, OVCAR4 and OV90 cells were double stained with anti-CD133/2-PE and anti-STn S3F-APC antibodies and sorted into STn^-^CD133^-^, STn^-^CD133^+^, STn^+^CD133^-^ and STn^+^CD133^+^ sub-fractions using the FACSAria flow cytometer (BD Biosciences). An aliquot of each sorted population was analyzed post- collection to confirm purity. The isolated sub-populations and non-sorted cells were resuspended in 0.6% agar and plated in the previously coated 6 well plates. After 21 days, colonies were visualized with nitroblue tetrazolium after overnight staining followed by methanol fixation and colonies comprising ≥20 cells were counted. Colony forming efficiency was determined as the (number of colonies formed/ number of cells plated) x 100.

### Sphere formation assay

OV90, OVCAR3, and OVCAR4 cells were plated in either their respective normal cell culture conditions or in ultralow attachment six-well plates (Sigma-Aldrich) at a density of 2 × 10^4^ cells/well in serum-free DMEM/F-12, HEPES medium with 1X B-27 supplement (Gibco), 10 ng/mL human epidermal growth factor (Life Technologies) and 20 ng/mL human β fibroblast growth factor (Life Technologies). Spheres were harvested after 10-12 days. Media was discarded and spheres were re-suspended in 0.25% trypsin. Cells plated under regular culture conditions as well as the sphere promoting conditions were stained for STn and CD133 and analyzed by flow cytometry as described.

### MTT cytotoxicity assay

OV90, OVCAR3 and OVCAR4 were seeded in 96-well plates, incubated overnight in the appropriate complete culture medium and then treated with increasing doses of the anti-STn-ADC S3F-CL-MMAE (0, 1.25, 2.5, 5, 10 and 20 nM) for 3 or 6 days (72 or 144 hours). Cell viability was determined by MTT assay and the percentage was calculated using the following formula: Percentage cell viability = (OD of the experiment samples / OD of the control) × 100.

### Evaluating STn levels post treatment *in vitro*

OV90, OVCAR3 and OVCAR4 were seeded in 6-well plates, incubated overnight in the appropriate complete culture medium and then treated with either 10 nM S3F-CL-MMAE or vehicle control. Cells were collected 3 or 6 days post treatment then assessed for STn and CD133 levels using flow cytometry and analyzed with FlowJo as described in the flow cytometry methods section.

### Extreme limiting dilution assay (ELDA)

OV90, OVCAR3 and OVCAR4 cells were plated in triplicate (3.5x10^5^ cells/well) in 6 well plates and treated after 24 hours with either 10 nM S3F-CL-MMAE or the vehicle control. After 3 or 6 days, cells were harvested, re-suspended in in serum-free DMEM/F-12, HEPES medium with 1X B-27 supplement (Gibco), 10 ng/mL human epidermal growth factor (Life Technologies) and 20 ng/mL human β fibroblast growth factor (Life Technologies) and plated in low adhesion 96 well plates at concentrations of 1, 10, 20 and 40 cells/well. After 7-8 days in culture, the wells were evaluated for presence or absence of tumorspheres. Tumor cell aggregates were not counted as positive unless individual tumorspheres were identified. Stem cell frequencies were then calculated as described (http://bioinf.wehi.edu.au/software/elda/).

### *In vivo* treatment studies

All mouse studies were carried out in compliance with our Institutional Animal Care and Use Committee guidelines. For generation of OVCAR3 cell line xenografts, 0.5 × 10^6^ cells were resuspended in PBS:Matrigel^®^ (1:1) and subcutaneously (s.c.) injected into 8-week-old female NOD/SCID mice (Jackson Laboratory, Bar Harbor, ME). All animals were monitored regularly for tumor formation and tumor volume was calculated using the formula (length x width x height)/2 as has previously been described [57]. When tumor volumes reached ∼200 mm^3^, the animals were randomized into 5 groups of 6-8 mice with equivalent average tumor volumes and subjected to one of five treatment regimens: (i) Vehicle control: intraperitoneal (IP) injection of 10 μM sodium citrate + 0.05% Tween 20 in sterile saline; (ii) IP injection of 5 mg/kg anti-STn antibody S3F (unconjugated) in sterile saline; (iii) IP injection of 5 mg/kg anti-STn-ADCS3F-CL-MMAE in sterile saline; (iv) IP injection of 5 mg/kg anti-STn antibody 2G12-2B2 (unconjugated) in sterile saline; (v) IP injection of 5 mg/kg anti-STn-ADC 2G12-2B2-CL-MMAE in sterile saline. All treatments were given weekly. Tumors were measured every 3 to 4 days with calipers and mice were weighed weekly. Six hours following administration of the last treatment, the mice were euthanized and the tumors harvested. Portions of each xenograft were formaldehyde-fixed and paraffin embedded for further analyses. The residual tumors from each group were pooled and processed to a single-cell suspension and stained for flow cytometric analysis of the STn population.

### Ki67 staining

OVCAR3 xenograft sections (5 μm) were examined for Ki67 expression levels by IHC. Antigen retrieval was performed in 10 mM sodium citrate buffer, pH 6.0, and slides were treated with 3% hydrogen peroxide. The blocking reagent and antibody diluent of a mouse-on-mouse (M.O.M.) kit (Vector Laboratories, Burlingame, CA) were used following the manufacturer’s instructions. Sections were incubated with a primary antibody against Ki67 (clone MIB-1, Dako), followed by incubation with an anti-mouse secondary antibody (M.O.M. kit, Vector Laboratories). Slides were then treated with Vectastain ABC reagents (Vector Laboratories) and further visualized using 3,30-diaminobenzidine chromogen (Dako, Carpinteria, CA). Sections with no primary antibody were used as negative controls.

### Statistical analysis

All experiments were carried out as 3-4 biological replicates with 3-4 technical replicates per iteration. Data were analyzed with GraphPad Prism 6.0 (GraphPad Software, La Jolla, CA). Bars represent mean ± SEM. One-way ANOVA was conducted to assess for significant differences in colony forming efficiency and response to STn-MMAE treatment. Two-way ANOVA was used to determine significant differences in population frequency following treatment with chemotherapeutics. The statistical significance of the observed differences in xenograft tumor growth and mouse weights between the different treatment groups was evaluated with two-way ANOVA. A *p* value < 0.05 was considered statistically significant.

## SUPPLEMENTARY MATERIALS FIGURES



## Abbreviations

STn: sialyl- Thomsen-nouveau
anti-STn ADC: STn-antibody drug conjugate
PBS: physiological buffered saline
PE: phycoerythrin
(ELDA): Extreme Limiting Dilution Assay
IP: intraperitoneal
S.C: subcutaneously
MTT: 3-(4,5-dimethylthiazol-2-yl)-2,5-diphenyltetrazolium bromide
IHC: immunohistochemistry
APC: allophycocyanin
NOD/SCID: nonobese diabetic-severe combined immunodeficiency
ATCC: American type culture collection
DMSO: Dimethylsulfoxide
