# Supplementary material for: Treatment of ovarian cancer by targeting the tumor stem cell-associated carbohydrate antigen, Sialyl-Thomsen-nouveau
Source: Oncotarget. 2018 May 1;9(33):23289–305. doi: 10.18632/oncotarget.25289 (PMC5955411; doi:10.18632/oncotarget.25289)
Supplement: Supplementary file 1 [file oncotarget-09-23289-s001.pdf]

Treatment of ovarian cancer by targeting the tumor stem cell-associated carbohydrate antigen, Sialyl-Thomsen-nouveau

SUPPLEMENTARY MATERIALS

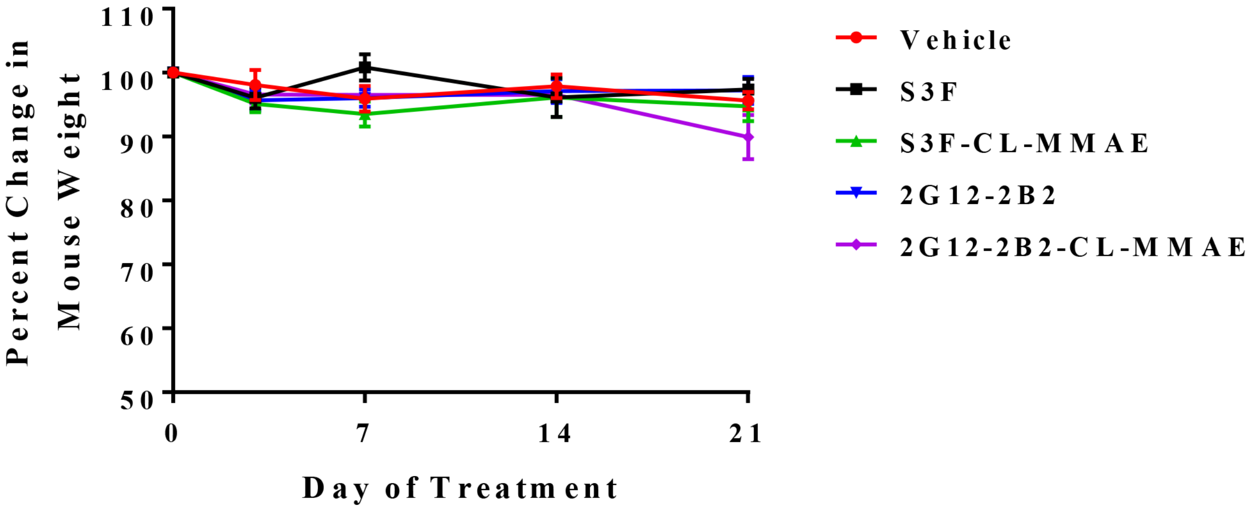

Supplementary Figure 1: *In vivo* treatment with anti-STn ADCs is well tolerated. Mice undergoing treatment *in vivo* with the unconjugated and MMAE-conjugated anti-STn antibodies were weighed every 4 days over the course of the treatment period. Percent change in mouse weight represents the change in weight relative to the weight at the beginning of the experiment. There was no significant weight loss in any cohort suggesting that the anti-STn ADCs are not toxic *in vivo*.

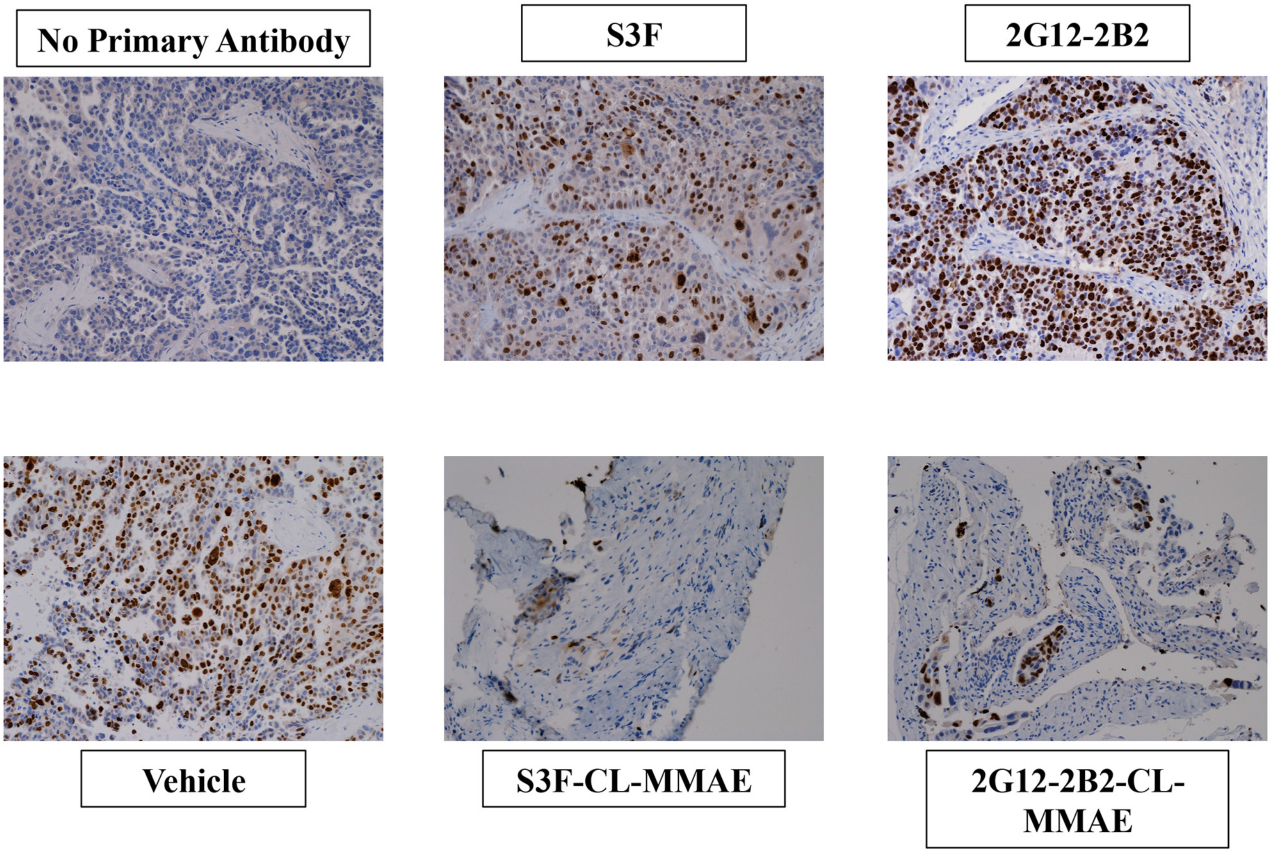

Supplementary Figure 2: Anti STn-ADCs reduce proliferation in treated tumors. A portion of each tumor harvested following *in vivo* exposure to unconjugated and MMAE-conjugated anti-STn antibodies was formalin fixed and paraffin embedded (FFPE). Slides generated from blocks representing each treatment cohort were subjected to Ki67 immunohistochemistry to assess qualitative differences in proliferating cells following treatment. Formalin-fixed paraffin-embedded (FFPE) xenograft sections were stained for the proliferative marker Ki67. Representative pictures of each staining are shown. Scale bar: 100 µm.
